# Advanced Crystallization Methods for Thin-Film Lithium Niobate and Its Device Applications

**DOI:** 10.3390/ma18050951

**Published:** 2025-02-21

**Authors:** Rongbang Yang, Haoming Wei, Gongbin Tang, Bingqiang Cao, Kunfeng Chen

**Affiliations:** 1 Institute of Novel Semiconductors, State Key Laboratory of Crystal Materials, Shandong University, Jinan 250100, China; 202484000296@sdu.edu.cn (R.Y.); gongbin.tang@sdu.edu.cn (G.T.); 2 Shandong Provincial Key Laboratory of Laser Polarization and Information Technology, School of Physics and Physical Engineering, Qufu Normal University, Qufu 273165, China; 3 School of Materials Science and Engineering, University of Jinan, Jinan 250022, China; mse_caobq@ujn.edu.cn

**Keywords:** LiNbO_3_ film, electro-optic modulation, epitaxial film, TFLN device

## Abstract

Lithium niobate (LiNbO_3_) has remarkable ferroelectric properties, and its unique crystal structure allows it to undergo significant spontaneous polarization. Lithium niobate plays an important role in the fields of electro-optic modulation, sensing and acoustics due to its excellent electro-optic and piezoelectric properties. Thin-film LiNbO_3_ (TFLN) has attracted much attention due to its unique physical properties, stable properties and easy processing. This review introduces several main preparation methods for TFLN, including chemical vapor deposition (CVD), molecular beam epitaxy (MBE), pulsed laser deposition (PLD), magnetron sputtering and Smartcut technology. The development of TFLN devices, especially the recent research on sensors, memories, optical waveguides and EO modulators, is introduced. With the continuous advancement of manufacturing technology and integration technology, TFLN devices are expected to occupy a more important position in future photonic integrated circuits.

## 1. Introduction

Lithium niobate (LiNbO_3_), as an excellent trigonal ferroelectric material [1], plays an important role in the field of electro-optics due to its unique crystal structure and outstanding physical properties [2]. Not only does the remarkable spontaneous polarization of LiNbO_3_ endow it with extremely strong ferroelectricity at room temperature [3], which makes LiNbO_3_ widely employed in ferroelectric devices, but also its electro-optic (EO) effect [4,5] and piezoelectric effect [6,7] show wide application potential in the fields of optical communication [8] and optical modulation [9]. In addition, the piezoelectric effect of LiNbO_3_ makes it widely used in sensors [10,11,12,13,14] and surface acoustic wave (SAW) devices [15,16,17]. With the rapid development of optical communication technology [18], the demand for high-performance optoelectronic devices is increasing [19,20]. Thin-film LiNbO_3_ (TFLN) has become a research hotspot due to its excellent optical, electrical and mechanical properties [21].

TFLN devices are developing toward high performance, low cost and multi-functionality. They play an important role in modern optoelectronics and quantum technology and have broad research and application prospects [22]. In this review, various preparation methods for TFLN (CVD, MBE, PLD, magnetron sputtering and Smartcut technology) are introduced in detail from two aspects of epitaxial thin films and single-crystal thin films, and the development status and future potential of TFLN devices (sensors, memories, optical waveguides and electro-optic modulators) are discussed. CVD technology has advantages in industrial production due to its high deposition rate and large-area uniformity [23]. In contrast, MBE technology shows great potential in the preparation of single-crystal films due to its precise atomic-level control and high-quality film growth [24,25]. PLD technology achieves uniform deposition on LiNbO_3_ substrates with various crystal orientations due to its flexibility and high efficiency [26], while the magnetron sputtering method is suitable for industrial production due to its low deposition temperature and accurate composition control [27]. Smartcut technology achieves the high-precision transfer of TFLN via ion implantation and bonding technology [28]. The development of TFLN devices, especially sensors, memories [29,30], optical waveguides [31,32,33,34] and electro-optic modulators [35,36,37] (EOMs), is driving the advancement of optical communication and microwave photonics. TFLN shows great advantages in the field of optical communication. For example, it is used to manufacture high-performance optical modulators, which can achieve higher modulation bandwidths, lower driving voltages and lower insertion losses and meet the needs of high-speed optical communication networks. These devices have become an indispensable part of photonic integrated circuits due to their advantages of miniaturization, high performance and integration [38]. Although several reviews describe methods for obtaining TFLN and thin-film-based devices, the current review is different. This review introduces various technical developments and optimization measures for the preparation of lithium niobate films, highlighting the optimization of the preparation methods for epitaxial lithium niobate films and their broad prospects in device applications. Over the background that the preparation of single-crystal thin films tends to be complete, the development of epitaxial thin films is undoubtedly an important means to reduce costs and improve performance.

## 2. Material Properties

LiNbO_3_ is a ferroelectric material belonging to the trigonal system. Its symmetry corresponds to the 3 m point group and the R3c space group. The crystal structure belongs to the ilmenite type [39] (ABO_3_) and is a typical distorted perovskite structure [40]. The ferroelectricity of LiNbO_3_ is derived from the polar arrangement of its crystal structure [41]. LiNbO_3_ has a non-centrosymmetric crystal structure at room temperature [42]. The asymmetric distribution of Li, Nb and O ions in the crystal structure leads to spontaneous polarization [43]. In the ferroelectric phase (trigonal system at room temperature), Li and Nb ions shift relative to the position of the oxygen ion octahedron to form spontaneous polarization. When the temperature is higher than the Curie temperature, LiNbO_3_ will transform into a paraelectric phase [44]. At this time, the crystal structure becomes a hexagonal system with higher symmetry, losing the spontaneous polarization characteristics. LiNbO_3_ exhibits considerable spontaneous polarization at room temperature. The polarization direction is along the c-axis of the crystal, and the polarization intensity is 50 μC/cm^2^. The ferroelectric hysteresis curve of LiNbO_3_ shows an obvious rectangular shape [45]. When the applied electric field is large enough, the polarization of the material can be completely reversed. When the reverse electric field is applied, the direction of spontaneous polarization can be reversed to form a new polarization state. The hysteresis behavior of the polarization–electric field relationship is a symbolic manifestation of the ferroelectric properties of LiNbO_3_, and its hysteresis characteristics are also related to the electrical memory effect [46].

The EO effect of LiNbO_3_ is closely related to its ferroelectricity. Since LiNbO_3_ exhibits spontaneous polarization, the applied electric field can affect its spontaneous polarization state, thus changing the refractive index of the crystal. By adjusting the applied electric field, the EO effect of LiNbO_3_ can perform an efficient optical modulation function [47]. The piezoelectric effect of LiNbO_3_ is also a direct result of its ferroelectricity [48]. Due to the existence of spontaneous polarization, the polarization will change under the action of external force, resulting in the inhomogeneity of charge distribution, resulting in the piezoelectric effect. This effect is widely used in sensors and SAW devices.

LiNbO_3_ is also an important photoelectric material. LiNbO_3_ has become one of the core materials of optical modulators, optical switches and phase modulators due to its significant EO effect [49]. The EO effect can be divided into the linear EO effect (Pockels effect) [50] and quadratic EO effect (Kerr effect) [51] according to its response to the electric field. The EO effect of LiNbO_3_ is mainly linear. This effect describes the phenomenon of the refractive index of LiNbO_3_ changing linearly under the action of an external electric field. The refractive index change is proportional to the size of the electric field [52]. This linear relationship causes the the EO modulation of LiNbO_3_ to have high response speed and high precision.

Since LiNbO_3_ crystal lacks inversion symmetry [53], this non-centrosymmetric structure enables it to produce a significant Pockels effect. The EO tensor of LiNbO_3_ describes the responsiveness of its refractive index to a change in the electric field. The material’s EO coefficient r_33_ is particularly prominent and is about 30.8 pm/V, making it efficient in EO modulation [54]. This means that under low-voltage conditions, LiNbO_3_ can produce significant refractive index changes, so it is suitable for optical devices that require low power consumption and high response speed [55]. Due to the extremely short response time of the Pockels effect of LiNbO_3_, it can complete the modulation of optical signals in nanoseconds or even picoseconds. Therefore, LiNbO_3_ is suitable for high-speed optical communication and ultra-fast laser technology [56]. This fast response characteristic is very important in modern communication systems. Especially with the increase in optical communication rate, the bandwidth requirements of modulation devices are also increasing. LiNbO_3_ has high thermal stability and can maintain stable electro-optical properties over a wide temperature range. Its Curie temperature is about 1210 °C, and the EO effect of LiNbO_3_ remains good at high temperatures, so it is suitable for applications that require high-temperature stability, such as aerospace and industrial optoelectronic devices [57,58].

## 3. Preparation Methods for TFLN

With the development of optical communication technology, the demand for high-performance optoelectronic devices is increasing. TFLN has become a research hotspot due to its excellent optical, electrical and mechanical properties. In this paper, several main preparation methods for TFLN are introduced in detail, including CVD, MBE, PLD, magnetron sputtering and Smartcut technology. The LiNbO_3_ films prepared by CVD, MBE, PLD and magnetron sputtering are epitaxial films, and the LiNbO_3_ films prepared by Smartcut technology are single-crystal films. TFLN prepared by Smartcut technology has been widely used in various LiNbO_3_ devices. Epitaxial TFLN shows broad development prospects.

An epitaxial film can precisely control its growth at the atomic level to obtain a high-quality crystal structure. Through epitaxial growth, a very clear and flat interface between the substrate and the film can be formed, which is crucial for the manufacture of high-performance semiconductor devices. Epitaxial growth can better control the stress in the film, which is very important for improving the mechanical stability and durability of the film. The type and concentration of dopants can be precisely controlled during the epitaxial growth process, which is very important for adjusting the electrical properties of the films. Epitaxial technology can be used to easily grow multilayer thin-film structures, which is very useful for manufacturing complex optoelectronic devices and quantum well structures. Although epitaxial films have these advantages, the preparation process usually requires higher technical requirements and more complex equipment, which may lead to increased costs. Therefore, in practical applications, it is necessary to choose a semiconductor film according to the specific requirements and conditions.

### 3.1. Chemical Vapor Deposition (CVD)

**Figure 1:**
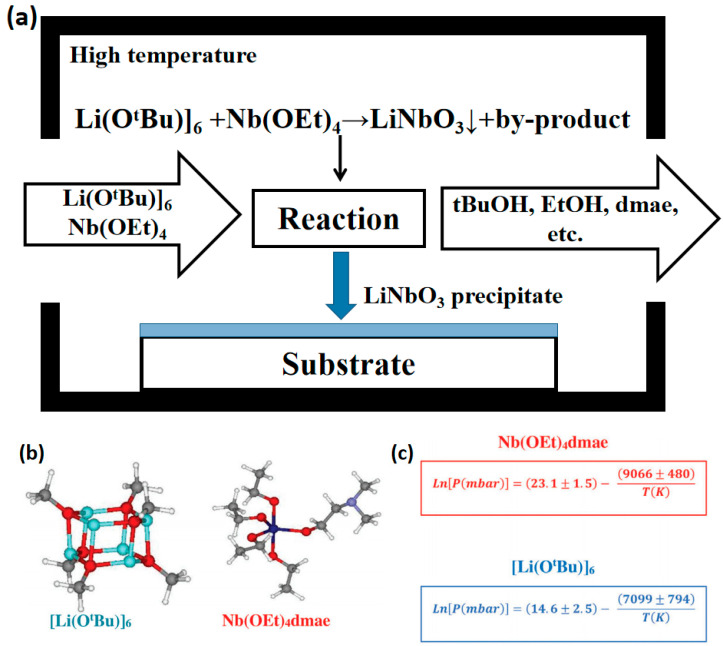
(**a**) Principle diagram of TFLN prepared by CVD. (**b**) Model structures of lithium and niobium precursors. (**c**) Pre-chamber vapor pressure equations for lithium and niobium precursors [62].

CVD is a thin-film growth method based on the gas-phase chemical reaction [59,60]. For LiNbO_3_ films, organometallic compounds containing niobium and lithium are usually used as precursors [61]. Using lithium tert-butoxide [Li(O^t^Bu)]_6_ and tetraethoxy dimethylamino ethoxy niobium Nb(OEt)_4_(dmae) as precursors in the high-temperature reaction chamber, the gas-phase precursor is decomposed, and LiNbO_3_ film is deposited on the surface of the substrate. The pre-chamber vapor pressure equations for lithium and niobium precursors are shown in Figure 1 [62]. The thickness and composition of the film can be precisely controlled by adjusting the flow rate of the reaction gas and the deposition time. The CVD deposition rate is high, and a large area of uniform film can be prepared, which is suitable for industrial production [63]. Using [Li(O^t^Bu)]_6_ and [Nb(OEt)_5_]_2_ as precursors, the growth rate is about 320 nm/h at a Li/Nb precursor ratio of 1:1 [64]. The film growth rate exceeds PLD and magnetron sputtering. It should be noted that due to the high toxicity of some precursors, they have a certain impact on the operating environment and film quality. Because CVD is sensitive to process parameters, it can easily produce defects, and the yield is low. The waste gas produced needs to be treated and discharged, so the preparation cost may be higher than that of the classical method. Table 1 introduces the precursors commonly used in the CVD preparation of TFLN and the optimization of preparation methods.

Lithium sources mainly include LiDPM, Li(O^t^Bu) and Li(thd), and niobium sources mainly include Nb(OEt)_5_, Nb(OEt)_4_(dmae) and Nb(thd)_4_. The volatility, thermal stability, deposition temperature and pressure of the precursor are optimized by the formation of bimetallic complexes, combined chemical methods, pulse-injection MOCVD and atmospheric-pressure MOCVD. The effects of precursor ratio on the phase composition, stoichiometric ratio, epitaxial quality and twin formation of the film are studied.

**Figure 2:**
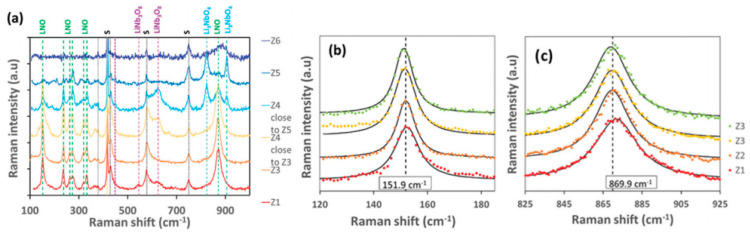
(**a**) Raman spectra of samples with different Li/Nb ratios. (**b**) Zoom at 150 cm^−1^. (**c**) Zoom at 870 cm^−1^ [62].

Anna L. Pellegrino et al. prepared TFLN using chemical beam evaporation deposition technology [62]. By changing the flow ratio of lithium and niobium precursors, the influence of this parameter on the quality of the film was systematically studied, as shown in Figure 2. The results showed that when the Li/Nb flow ratio is close to the stoichiometric ratio, the prepared LiNbO_3_ film exhibits excellent crystalline quality, including a single phase, low inlay, low surface roughness and refractive index and band gap close to the bulk. In addition, using RF glow discharge emission spectroscopy, the researchers screened the film composition quickly and effectively for the first time to reflect the crystal quality at different Li/Nb ratios. The research results are of great significance for the development of high-performance modulators and integrated photonic devices in mobile communication and other fields.

CVD technology can accurately control the thickness and composition of thin films and has been widely used in the preparation of high-quality TFLN [68]. Previous studies mainly focused on optimizing deposition parameters (such as temperature, gas flow rate and precursor ratio) to achieve high uniformity and low defect density in single-crystal films. At present, epitaxial LiNbO_3_ films can be successfully deposited on sapphire substrates by solid-source CVD technology. The films exhibit excellent crystal quality and optical properties, which are suitable for optical and acoustic waveguide devices [69]. New precursors have been developed to improve the deposition of thin films during CVD [61,70]. The sol–gel spin-coating method using a heterogeneous lithium–niobium precursor has been shown to improve the uniformity and surface smoothness of the film [71]. In addition, this method further improves the physical properties of the film during low-temperature deposition, such as its thermal stability and dielectric properties [72]. The innovation of these precursors not only improves the performance of the film but also reduces the energy consumption during the preparation process and makes it more environmentally friendly. In recent years, the application of CVD technology in the preparation of TFLN has made great progress. By optimizing the deposition conditions and introducing new precursors, not only has the quality of the film been successfully improved, but also new application fields have opened up [73]. However, CVD technology usually requires the use of organometallic compounds as precursors. These precursors are often highly toxic and have adverse effects on the operating environment and film quality, requiring strict exhaust gas treatment measures. Reducing environmental pollution and developing precursors with low toxicity and high stability is one of the focuses of current research.

### 3.2. Molecular Beam Epitaxy (MBE)

**Figure 3:**
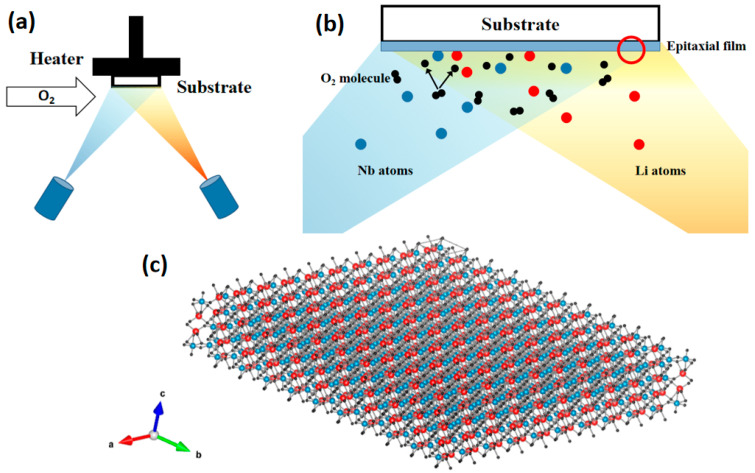
(**a**) Schematic diagram of TFLN prepared by MBE. (**b**) Reaction diagram at the substrate surface. (**c**) Atomic arrangement diagram of TFLN.

MBE is a method of forming thin films by depositing a pure metal or compound on a heated substrate in the form of atoms or molecular beams in an ultra-high-vacuum environment [74,75,76,77]. For TFLN, the source materials of niobium and lithium generate molecular beams by electron beam heating, and the beam current is precisely controlled so that the growth of thin films has high quality and high precision [24,74]. The basic principle of MBE is shown in Figure 3. The substrate is fixed in the vacuum chamber, and the vacuum degree is kept below 10^−9^ Torr. The molecular beam source furnace heats the source materials of niobium and lithium and emits them to form an atomic beam. Oxygen is introduced around the substrate. On the surface of the substrate, Nb and Li atoms interact with oxygen to form a LiNbO_3_ film. By controlling the substrate temperature, evaporation rate and oxygen flow rate, the quality of the film is ensured [78]. Its advantage is that it can achieve precise control at the atomic level, and the film quality is extremely high. It is suitable for preparing high-quality single-crystal films. It can monitor the film growth in real time during the deposition process and has good controllability. Table 2 introduces commonly used precursors for the preparation of TFLN by MBE and the optimization of preparation methods.

In the early stage of MBE development, niobium and lithium were used as precursors, and researchers later gradually turned to a LiNbO_3_ target or to niobium pentachloride as a niobium source and lithium as an auxiliary material. Over time, the optimization of precursors has evolved from simple electron beam evaporation to L-MBE, and oxygen free-radical sources and lithium capture agents have been introduced to improve reactivity and control stoichiometry. From the double-cavity system to low-background-pressure, to high-temperature growth, the optimization of the instrument is mainly focused on improving the vacuum environment, controlling the growth temperature and introducing in situ monitoring technology to ensure the high-quality growth of the film.

**Figure 4:**
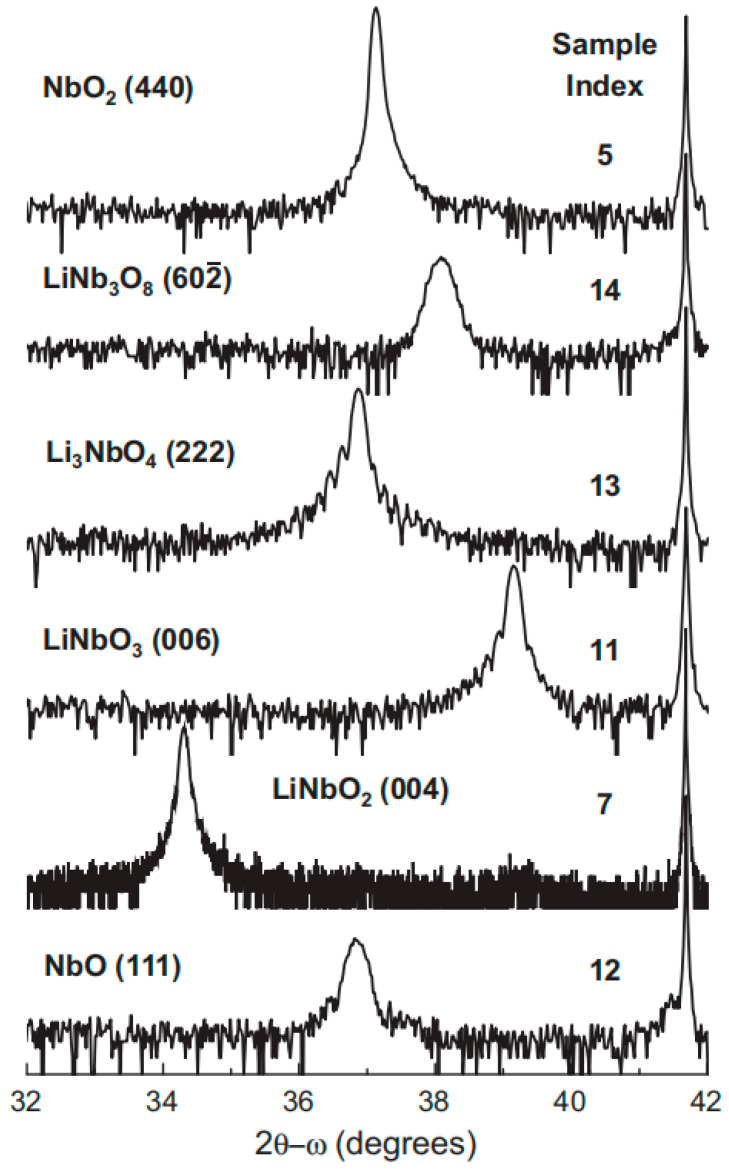
Growth of a single-phase Nb-Li-O structure on c-axis alumina. Reprinted/adapted with permission from Ref. [80]. 2017, Elsevier.

Brooks Tellekamp et al. studied the effects of the stoichiometric ratio and growth temperature of lithium niobium oxide (Li-Nb-O) on the phase-selective nucleation of materials by MBE and constructed an empirical growth phase diagram [80]. They found that although a single-parameter change usually produced a multiphase film, combining substrate temperature control with the previously published lithium flux-limited growth method allowed for the repeated growth of a variety of high-quality single-crystal films with different oxide phases [81]. A higher temperature (800–1050 °C) was required to achieve the growth of high-quality materials compared to the temperature commonly used in MBE [82]. Single-phase films of NbO, NbO_2_, LiNbO_2_, Li_3_NbO_4_, LiNbO_3_ and LiNb_3_O_8_ were successfully grown on c-plane sapphire using this method, as shown in Figure 4. This study not only optimizes the growth of Li-Nb-O materials in a single deposition system but also realizes the controllable growth of single-phase thin films with different oxide phases by accurately controlling the stoichiometric ratio and growth temperature, which provides important experimental data and theoretical support for the design of new electronic devices and materials. In addition, a Li-Nb-O phase diagram based on the experimental results is proposed, which guides the growth of these materials on sapphire substrates.

In recent years, oxygen radical-assisted laser MBE (ORA-LMBE) technology has been used to produce TFLN, which significantly improves the crystal quality of the films [74]. This method effectively prevents the volatilization of Li by introducing high-energy oxygen radicals and helps to form high-quality single-crystal films. Compared with the traditional MBE method, ORA-LMBE can generate films with higher crystal quality at lower growth temperatures while maintaining the stoichiometry and lattice integrity of the films [83]. In addition to traditional MBE and ORA-LMBE, researchers have also developed MBE processes using chloride precursors [84]. These methods significantly shorten the production cycle by increasing the growth rate while maintaining the high crystal quality of the film. Although this method has achieved results comparable to those of bulk materials in crystal quality, the growth rate is relatively slow, and further optimization is still needed to adapt to large-scale industrial production.

However, a high substrate temperature (800 °C) is usually required for the preparation of TFLN by MBE. This high-temperature condition may lead to thermal damage of the substrate material and also puts forward higher requirements for the stability of the growth equipment. Lithium is easy to volatilize at high temperature, which may cause the composition of the film to deviate from the stoichiometric ratio to form a multiphase structure (NbO, NbO_2_, LiNbO_2_, etc.), affecting the crystal quality and electrical properties of the film. The accurate control of growth parameters (substrate temperature, lithium/niobium flow ratio) is the key to achieve single-phase thin-film growth. With the introduction of oxygen radical-assisted MBE technology and the development of new precursor materials, the crystal quality and growth rate of TFLN have been significantly improved. However, the application of the MBE process in large-scale production still needs to be further explored to meet the needs of modern integrated optoelectronic devices.

### 3.3. Pulsed Laser Deposition (PLD)

**Figure 5:**
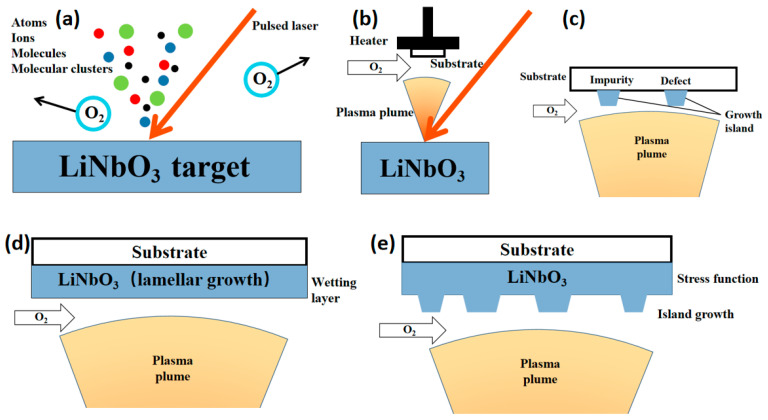
(**a**) A laser bombards the surface of the target to produce atoms, molecules and clusters. (**b**) The generated plasma plume reaches the substrate surface. (**c**) The growth island is preferentially formed in the defects and impurities on the substrate surface. (**d**) The plasma continues to grow along the growth island to form a LiNbO_3_ film. (**e**) Due to the lattice constant difference under stress, the LiNbO_3_ film is a single-crystal film with island growth.

PLD is a technology in which targets are bombarded by a high-energy pulsed laser beam, and the target is evaporated and deposited on the substrate to form a thin film [85,86]. The PLD preparation of TFLN usually uses single-crystal LiNbO_3_ as the target and sapphire as the substrate. The principle is shown in Figure 5. The LiNbO_3_ target is placed in the vacuum chamber, and the target is bombarded by a high-energy pulsed laser to produce atoms, molecules, ions, molecular clusters, etc., which continue to absorb laser energy and heat up sharply to form a plasma plume [87]. After isothermal expansion and adiabatic expansion, the plume reaches the surface of the substrate and deposits the defects and impurities on the surface of the substrate to form a growth island in an oxygen-rich atmosphere [88]. Subsequent deposition diffuses around the growth island to form a LiNbO_3_ film. The initial film grows layer by layer. Subsequently, due to the difference in lattice constants, island growth begins under stress. PLD technology can accurately control the quality and thickness of the film by adjusting the substrate temperature, laser pulse frequency, target rotation and other parameters to achieve epitaxial growth. By adjusting the composition ratio of the target, the required film with the same composition as the target can be deposited [89,90]. Due to the slow film growth rate of this method, it is difficult to prepare large-area films and realize large-scale industrial production.

In the past two years, PLD technology has made remarkable progress in the preparation of TFLN [26,91]. PLD technology has attracted much attention because it is suitable for the preparation of thin films with high-quality and complex oxide materials [92]. At present, high-quality LiNbO_3_ films can be successfully grown on sapphire substrates by PLD [93]. The deposition conditions of the thin film are continuously optimized, which reduces the generation of the Li defect phase. Different from CVD and MBE, the target used in PLD is almost constant. By directly using a lithium niobate target for sputtering growth, the ratio of Nb and Li in the target can be changed with the requirements. Table 3 introduces the optimization of TFLN prepared by PLD.

The use of an Li-enriched target can ensure the stoichiometric ratio of the film and reduce Li loss. The optimization of thin-film growth conditions is focused on the following:

1. Laser: Laser energy density and pulse frequency are optimized in order to improve the quality and uniformity of the film.
2. Gas environment: Appropriate oxygen pressure can effectively reduce oxygen vacancies in the film and reduce the generation of an anoxic phase.
3. Substrate temperature: The substrate temperature is usually controlled at 500–800 °C to promote the crystallization of the film and reduce Li loss.
4. Annealing treatment: Annealing treatment is performed after deposition to reduce oxygen vacancies and improve the optical properties of the film.

**Figure 6:**
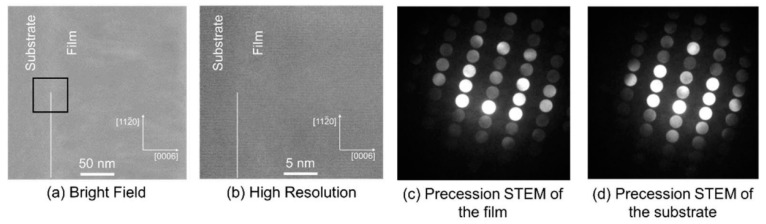
(**a**) Bright-field STEM image. (**b**) High-resolution image of an epitaxial LiNbO_3_ Z-cut film on a Z-cut LiNbO_3_ substrate. (**c**) Precession STEM of the sample layer. (**d**) Precession STEM of the substrate. Reprinted/adapted with permission from Ref. [26]. 2023, Elsevier.

Laura C. Sauze et al. successfully grew high-quality TFLN on LiNbO_3_ substrates with different crystal orientations (X-cut, Y-cut and Z-cut) using PLD technology [26]. A high-resolution X-ray diffraction (HRXRD) method was developed to characterize the crystal properties of these homoepitaxial layers. As shown in Figure 6, the interface between the film and the substrate on the Z-cut substrate was observed by transmission electron microscopy (TEM), and it was found that the interface was almost defect-free. The results show that the crystal quality and stoichiometry of TFLN grown by PLD technology are very close to those of single-crystal materials. In addition, the study also revealed that the chemical composition of the film is related to the crystal-cutting method of the substrate, and the film grown on the Y-cut substrate shows different composition characteristics from other crystal orientations. This study provides a new method to precisely control the growth and properties of thin films, which is of great significance for the development of next-generation high-performance RF filters.

Through PLD technology, uniform deposition can be achieved on LiNbO_3_ substrates with various crystal orientations, and the prepared films have excellent structural and optical properties. In addition, the LiNbO_3_ film prepared by PLD can also be applied to develop film bulk acoustic resonators [99] (FBARs) and have great potential for high-frequency applications, especially in 5G and wireless communication technologies [100]. The microstructure quality of the film and the quality factor of the resonator can be significantly improved by further optimizing the electrode material and deposition conditions. Although PLD technology has the advantages of high precision and strong controllability, the film growth rate is usually slow, the deposition area is small and it is difficult to achieve the rapid preparation of large-area films, which limits its application in large-scale industrial production. PLD technology shows great flexibility and efficiency in the preparation of TFLN. In future research, the further optimization of deposition parameters and the exploration of new substrate materials will help to promote the wide application of TFLN in optoelectronic devices, acoustic filters and nonlinear optics.

### 3.4. Magnetron Sputtering Method

**Figure 7:**
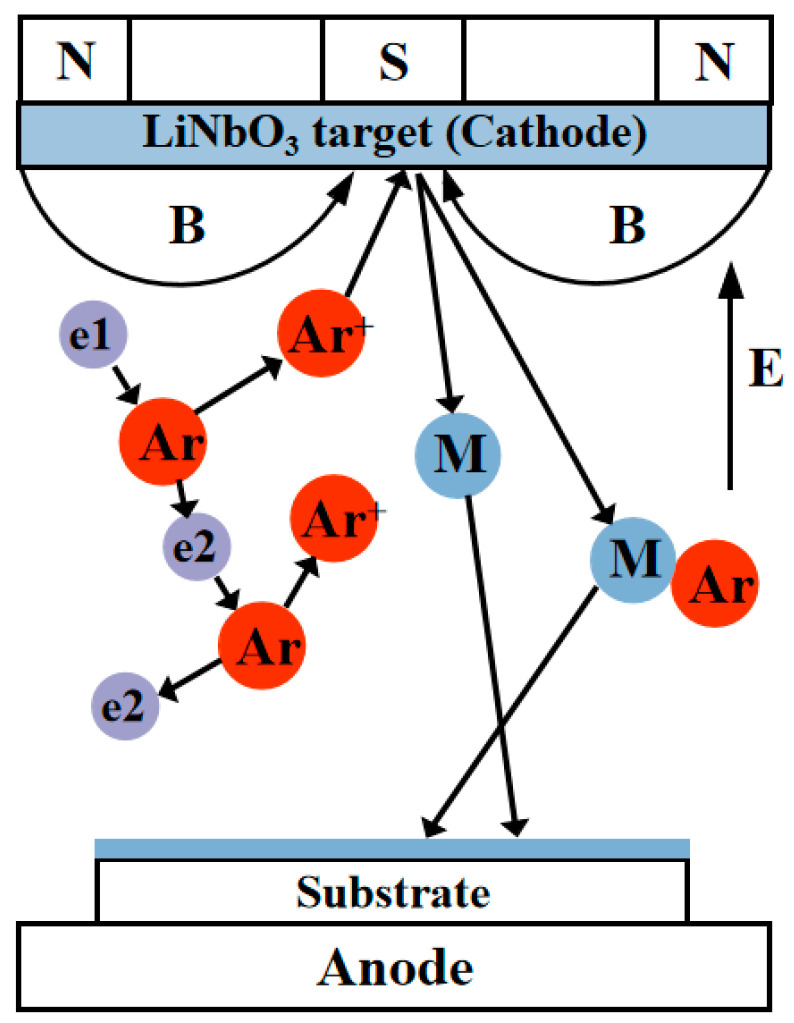
Principle diagram of TFLN grown by magnetron sputtering.

Magnetron sputtering is a physical vapor deposition technology that uses high-energy ions to bombard the target and cause the target atoms to sputter onto the substrate to form a thin film [101,102]. The principle of magnetron sputtering is shown in Figure 7. The substrate and the target are at the anode and cathode, respectively, and the direction of the electric field generated is shown with label E in Figure 7. The back of the target is a permanent magnet, which produces a strong magnetic field orthogonal to the electric field. Under the action of electric field E, the electrons accelerate to the substrate at the anode position, collide with the argon atom and ionize the argon atom to produce Ar^+^ and new electrons (secondary electrons). Due to the positive charge of Ar^+^, the target with high negative pressure is accelerated under the action of the electric field, so that the target is sputtered, and the sputtered target atoms are finally deposited on the substrate to form a film. Meanwhile, electrons in the plasma are influenced by both the electric field force and the Lorentz force. The spiral motion around the magnetic field line near the target is greatly prolonged, and the probability of collision with the argon atom is greatly increased. As a result, more Ar^+^ ions bombard the target through these collisions, thereby significantly enhancing the sputtering rate.

Magnetron sputtering can deposit thin films at lower temperatures, reduce the thermal damage of substrate materials and control the deposition thickness and film composition more accurately [103]. Compared with PLD technology, the film area is larger, which is suitable for industrial production. Due to the sputtering characteristics of the target, the film composition may deviate during the deposition process, and the process parameters need to be adjusted for compensation. Table 4 introduces the measures for optimizing the quality of the film and for the optimization of the preparation method of the instrument from several studies on the preparation of LiNbO_3_ films by magnetron sputtering.

Ar/O_2_ mixed gas is usually used to prepare LiNbO_3_ films by magnetron sputtering. Appropriate gas pressure and ratio can effectively reduce oxide reduction and oxygen vacancies. The substrate heating temperature is usually controlled at 380–550 °C to promote the crystallization of the film and reduce Li loss. Through the multi-step sputtering process, the film thickness is gradually increased to ensure that the film has good structural and optical properties.

Luying Yin et al. studied the third-order nonlinear optical properties of Nb-rich TFLN prepared by magnetron sputtering at room temperature and annealed at 700 °C in high-purity oxygen [107]. The TFLN exhibited reverse saturation absorption and self-focusing behavior. The third-order nonlinear refractive index γ and absorption coefficient β were determined to be about 10^−11^ cm^2^/W and 10^−7^ cm/W, respectively. The γ value was four times larger than that of single-crystal LiNbO_3_ measured at a 532 nm wavelength and 25 ps pulse width, while the γ value was two orders of magnitude smaller than that of LiNbO_3_ films measured at 532 nm and 800 nm wavelengths. The nonlinear absorption of another LiNbO_3_ film was significantly lower than that of the previously reported LiNbO_3_ film [108]. The results show that the optical nonlinearity of TFLN was significantly affected by atomic composition and incident wavelength. By changing the atomic composition and test conditions of the thin films, significant changes in optical nonlinearity were observed, which is of great significance for LiNbO_3_-based integrated photonic devices.

At present, magnetron sputtering can be used to successfully prepare high-quality TFLN on sapphire and other substrates by using a multi-step sputtering process [104,106]. The film has excellent epitaxial characteristics. The main advantage of this multi-step process is to control the thickness of the thin film on the premise of maintaining a good epitaxial structure and reducing interface defects, thereby improving optical and electrical properties. By improving the sputtering process, the film exhibits an excellent piezoelectric response in a wide frequency range [7]. By accurately controlling the sputtering parameters and substrate temperature, the piezoelectric coefficient of the LiNbO_3_ film can be significantly improved, which is of great significance for the future development of high-performance sensors, transducers and MEMS devices.

Although magnetron sputtering technology has made significant progress in the preparation of TFLN, there are still some challenges to be solved. Magnetron sputtering technology is usually employed at a lower temperature, which often leads to insufficient crystallinity in the film. Subsequent annealing treatment can effectively improve the crystal quality of the film. During the sputtering process, the sputtering rates of lithium and niobium are different, which easily leads to the deviation of the film composition from the stoichiometric ratio and affects the crystal quality and properties of the film. At the same time, it is also necessary to accurately control the oxygen flow rate. Insufficient oxygen content may lead to the formation of oxygen vacancies, affecting the electrical and optical properties of the film. Although magnetron sputtering is suitable for large-area film preparation, it struggles achieve uniform film thickness on a large-area substrate due to the influence of the target glow position and plume diffusion. It is still necessary to further improve the sputtering parameter control to reduce the defect density and improve the uniformity of the film. For example, a multi-pole magnetic field design is used to optimize the magnetic field distribution and reduce the inhomogeneity of the magnetic field by adjusting the position and intensity of the magnetic pole. Rotating the target and substrate can also improve the uniformity of the film.

### 3.5. Smartcut Technology

Smartcut technology is an advanced thin-film material preparation technology that was applied to silicon-based materials in its early stages and has been successfully applied to the preparation of TFLN [109]. The basic principle of Smartcut technology is to strip a high-quality LiNbO_3_ film from a large LiNbO_3_ crystal and transfer it to another substrate through a series of processes such as ion implantation, bonding and stripping [110]. The obtained lithium niobate thin film has a high transmittance and electro-optic coefficient, and the number of defects (such as dislocations, grain boundaries, microcracks, etc.) in the crystal is very small. First, a high-concentration ion layer is formed on the shallow surface of the LiNbO_3_ crystal by the ion implantation process (usually hydrogen or helium ions), and the expansion of the ion layer produces microcracks. Next, the ion-implanted LiNbO_3_ wafer is bonded to another substrate (such as a silicon or oxide substrate) by high-temperature annealing so that the LiNbO_3_ film is firmly attached to the substrate. Finally, with the effect of temperature and mechanical stress, the ion implantation layer guides the surface of the wafer to produce precise cracks, thus stripping out a layer of film [111]. This method can control the thickness of the film with high precision, and the stripping process is relatively mild and will not destroy the crystal structure of the film.

The preparation of TFLN using Smartcut technology has significant advantages. First, it can precisely control the thickness of TFLN, which is crucial for the design and performance optimization of optical devices. The thickness accuracy of the film directly affects the performance of optoelectronic devices such as waveguides and modulators. Second, Smartcut technology enables the transferred LiNbO_3_ film to have a high-quality crystal structure and low surface roughness by means of ion implantation and mechanical stripping, which can maintain the optical and electro-optical properties of the original material [112]. In addition, TFLN can be transferred to a variety of substrates, especially silicon substrates, achieving compatibility with existing silicon-based integrated circuit technologies [113]. This feature is of great significance for the development of optoelectronic integrated circuits, making it possible to integrate photonic and electronic functions on a single chip.

However, the application of Smartcut technology in the preparation of TFLN is also insufficient. The ion implantation process makes it easy to introduce defects, which may affect the electro-optical properties of TFLN and the long-term stability of materials. The steps of ion implantation, bonding and stripping require high equipment and process control, resulting in high production costs. At present, Smartcut technology is still the most efficient way to prepare TFLN on a large scale. Reducing production costs and optimizing processing technology to improve film quality are problems that need to be solved in the commercial application of TFLN.

Table 5 summarizes the advantages and disadvantages of different TFLN preparation methods and the subsequent optimization directions. At present, the preparation of TFLN wafers still faces limitations of size and yield. In the future, it is necessary to improve the size and yield of the wafer by optimizing the process flow and improving machining accuracy. This is an important development direction to improve large-area epitaxial film preparation and further optimize the integration density of TFLN photonic integrated circuits while maintaining high performance.

## 4. TFLN Devices

As an important ferroelectric, piezoelectric and photoelectric material, LiNbO_3_ has excellent EO, acousto-optical and nonlinear optical properties and can effectively modulate optical signals [114]. Therefore, it has very important application prospects in optical communication and microwave photonics. Based on the requirements of industrial production, most of the TFLN currently used in these devices is prepared by Smartcut technology. The preparation of TFLN by this technology has matured. Moreover, the LiNbO_3_ single-crystal film has a large area and good uniformity, which is suitable for device requirements. It is worth mentioning that the LiNbO_3_ epitaxial films prepared by PLD, CVD and other methods also show some advantages. Although large-area production is still an important problem, epitaxial film growth is a good method to further optimize the device performance. With the development of thin-film preparation technology, TFLN devices have become a research hotspot. This section will focus on common TFLN devices.

### 4.1. TFLN Sensors

At present, TFLN has been widely used in sensors [115,116]. Based on its EO effect and piezoelectric effect, it can be used to detect electric fields, magnetic fields, temperature, pressure and other physical qualities [117,118,119]. Such sensors usually have high sensitivity, fast responsiveness and good stability. The basic working principle of TFLN optical sensors depends on the EO effect and piezoelectric effect of the material. In an EO effect sensor, when an external electric field is applied to the LiNbO_3_ film, the refractive index of the material will change, which will affect the propagation characteristics of the light wave passing through the film [120]. The magnitude and direction of the external electric field can be determined by monitoring changes in the optical signal. In sensors based on the piezoelectric effect, the application of external pressure or mechanical stress to the LiNbO_3_ film induces a redistribution of positive and negative charges within the material. This redistribution, in turn, affects the electrical or optical properties of the material, allowing the sensor to detect external stress or pressure through these alterations. Optical sensors are usually combined with waveguide technology to improve the sensitivity of optical signal detection through integrated waveguide structures.

**Figure 8:**
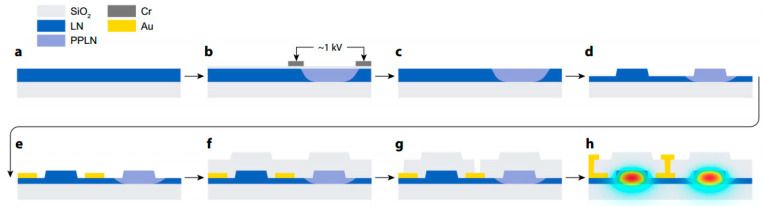
Fabrication process of a quantum optical phase sensor [14]. (**a**) Starting from the 500 nm film LN on the insulator. (**b**) In order to periodically pole the LN film, a chromium electrode deposited on a 100 nm silica insulating layer was used. A high voltage pulse is applied to reverse the crystal domain. (**c**) Chromium electrode and silica layer were removed after polarization using chromium etching solution and buffer oxide etching solution. (**d**) Waveguides are fabricated by electron beam lithography and argon ion grinding. (**e**) A layer of gold is directly covered on LN to increase the electric field in the waveguide region. (**f**) The waveguide covers about 700 nm of silicon dioxide. (**g**) Open the vias in the cladding to provide electrical contact with the embedded electrode. (**h**) covers the top gold to connect the device and the external probe.

Hubert S. Stokowski et al. [14] introduced a quantum optical phase sensor integrated on a LiNbO_3_ film, as shown in Figure 8. An X-cut lithium niobate film on insulator (LNOI) was prepared using Smartcut technology. The sensor uses the quantum noise limit of light to achieve phase detection sensitivity beyond the quantum noise limit by generating a squeezed state with the same frequency as the pump light. The photonic system integrates all the necessary functions, such as generating the pump light, locking the phase and realizing interferometer measurements, producing an integrated quantum sensor on a single chip. With the characteristics of low power consumption and monolithic integration, the sensor offers new opportunities for the development of quantum optical sensing and provides a promising method for the development of quantum-enhanced optical sensors. Epitaxial LiNbO_3_ films can achieve higher surface acoustic wave velocity, which is expected to provide higher detection sensitivity. Methods to obtain a large area of uniform epitaxial film still need continued attention.

TFLN sensors have the advantages of high sensitivity and fast response. The EO coefficient of LiNbO_3_ material is large. The sensors can detect weak electric fields or stress change with very high sensitivity and respond quickly to external signals, which is suitable for real-time monitoring [121]. At present, the factor that affects their industrial production is the complex manufacturing process. The preparation of TFLN sensors requires high-quality thin-film deposition and waveguide processing technology, which makes the manufacturing cost high, especially in large-scale production.

### 4.2. TFLN Domain Wall Memory

LiNbO_3_ domain wall memory is a new type of memory based on the domain structure and domain wall movement characteristics of LiNbO_3_ materials [122]. LiNbO_3_ is a typical ferroelectric material. There are spontaneous polarization regions inside the crystal. These regions are called domains [123]. The interface between domains is called a domain wall. Domain walls have special physical properties and can be controlled and moved by an external electric field or stress [124]. In domain wall memory, the storage and reading of information are achieved by manipulating the domain wall position and its motion in LiNbO_3_.

LiNbO_3_ has strong ferroelectricity, and its domain structure can exist stably at the micro-scale [125]. In ferroelectric materials, the formation of domains is due to the spontaneous arrangement of internal electric dipole moments, and these dipole moments will form polarization regions in different directions inside the material. Due to its unique crystal symmetry and extremely high Curie temperature, the domain structure of LiNbO_3_ is extremely stable and can be maintained at room temperature for a long time [126]. The domain wall is an interface where the polarization direction is reversed. This interface often has physical and chemical properties different from those of the material body, especially in terms of electrical, optical and mechanical responses [127].

The principle of domain wall memory mainly depends on the precise control of the domain wall position. By applying an external electric field to the LiNbO_3_ crystal, the domain wall can be moved. Alternatively, local heating and stress can be used to alter the position and shape of the domain wall. The movement of the domain wall is used to represent different storage states, and the storage of binary information is realized by manipulating the position of the domain wall [128]. A key advantage of domain wall memory is its high stability of information storage. Since the ferroelectric domain of LiNbO_3_ material has a highly stable polarization state, the information can be maintained for a long time without loss, similar to the traditional non-volatile memory. This allows the LiNbO_3_ domain wall memory to retain stored information after a power failure, similar to flash memory. Compared with traditional volatile memory, domain wall memory does not require a continuous power supply to maintain information, which allows it to exhibit extremely low power consumption, especially suitable for low-power-consumption and long-life application scenarios [129]. In addition, the EO effect and nonlinear optical properties of LiNbO_3_ material provide a unique optical reading method for domain wall memory. Since the refractive index of LiNbO_3_ material changes with the change in electric field, the optical signal can be guided to the LiNbO_3_ crystal by an optical waveguide or optical fiber, and the storage state can be read by detecting the change in the domain wall. This optical reading method can not only achieve high-speed data reading but also avoid losses in the transmission of electrical signals and improve reading efficiency.

**Figure 9:**
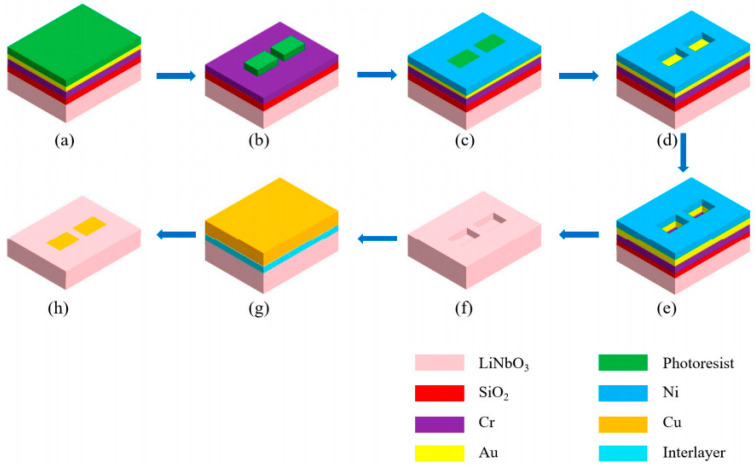
Fabrication processes for LiNbO_3_ memory devices [30]. (**a**) SiO_2_, Cr and Au were deposited on the surface of Au layer as A seek layer and spin-coated photoresist. (**b**) The bare marker area after EBL patterning. (**c**) Electroplating Ni layer on the seed layer without photoresist. (**d**) Remove photoresist. (**e**) Au, Cr, SiO_2_ and LiNbO_3_ were etched by reactive ion etching. (**f**) Removal of Ni, Au, Cr and SiO_2_. (**g**) Deposition of interlayer and Cu. (**h**) Cu outside the groove area was removed by chemical–mechanical polishing.

Haiqing Jiang et al. [30] proposed a method to improve the performance of LiNbO_3_ domain wall memory by optimizing the metal–semiconductor contact, as shown in Figure 9. The device is fabricated from X-cut lithium niobate by CVD and magnetron sputtering. By introducing a 10 nm interlayer between the LiNbO_3_ and the copper electrode, the domain wall current density and selectivity of the device are significantly improved. The writing time of the device with titanium (Ti) intercalation is 82 ns at a voltage of 8 V, and the erasing time is 12 µs, which is much faster than that of the device without intercalation. In addition, the improved device shows a retention time of more than 10^6^ s and a switching ratio of 10^5^ at a high temperature of 400 K. These intercalations not only reduce the coercive voltage of the device but also increase the domain wall current. At the same time, they effectively suppress the imprinting effect and improve the stability and reliability of the device. This work provides an important technical basis for the realization of high-density, high-performance domain wall random access memory. It is worth mentioning that the LiNbO_3_ epitaxial film prepared by PLD technology exhibits island-like growth, and the film is stacked with dense growth islands. This is more conducive to the formation of a thin-film ferroelectric domain structure, which may be more suitable for domain wall memory.

LiNbO_3_ domain wall memory has broad application prospects for the future. Its non-volatility and low power consumption characteristics make it very suitable for mobile devices, Internet of Things and edge computing scenarios. These applications usually require devices to maintain data integrity under low power consumption or no power supply. Second, due to the unique electro-optical properties of LiNbO_3_ materials, domain wall memory can also be integrated with photonic devices to develop optoelectronic hybrid memory [130]. These devices can achieve fast and efficient conversion between optical signals and electrical signals and can be directly combined with photonic integrated circuits. This technology plays an important role in the next generation of optical communication networks.

### 4.3. TFLN Waveguides

LiNbO_3_ is an ideal material for optical waveguide devices due to its excellent EO and nonlinear optical properties [34,131]. The basic structure of a TFLN optical waveguide is composed of TFLN as the core waveguide layer, a cladding layer and a substrate. The optical signal propagates through the waveguide layer, and the difference in the geometric structure of the waveguide and the refractive index of the material determines the propagation mode and speed of the light passing through it [33]. By applying an external electric field, the refractive index of the LiNbO_3_ film can be modulated, thereby controlling the phase and propagation path of the optical signal. TFLN optical waveguide devices are often used in combination with other optoelectronic devices such as optical splitters, optical modulators [132] and optical filters [133]. TFLN optical waveguide devices play a central role in integrated photonic circuits.

**Figure 10:**
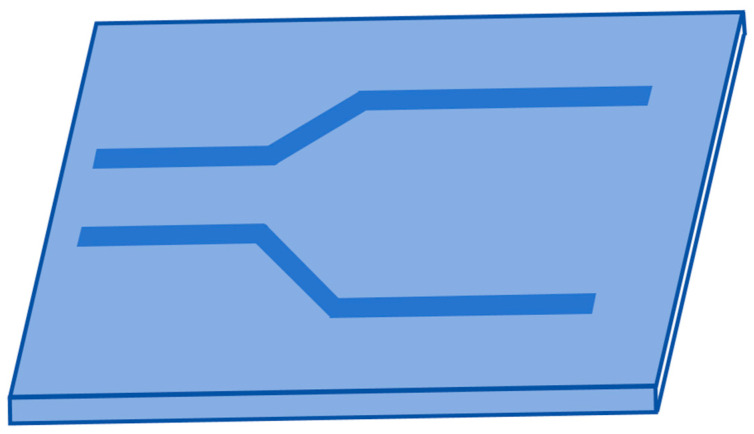
Schematic structure of a 3-dB coupler.

The EO effect of LiNbO_3_ enables the waveguide device to achieve fast optical signal modulation. The film has a low optical loss and can ensure the efficient transmission of optical signals in the waveguide. In addition, LiNbO_3_ has a wide optical transparent window (from ultraviolet to mid-infrared), so TFLN optical waveguide devices can work in a wide wavelength range [134]. Wen-Hung Huang et al. [131] introduced a new method for fabricating LiNbO_3_ optical waveguides using gallium oxide as a diffusion source. The waveguide was fabricated on a Y-cut LiNbO_3_ substrate using Smartcut technology, and the waveguide pattern was constructed by lithography and magnetron sputtering. The diffused gallium was analyzed by secondary ion mass spectrometry (SIMS), and its optical waveguide characteristics were studied. The waveguide had low propagation loss at wavelengths of 632.8 nm and 1550 nm and could achieve single-mode single-polarization transmission. In addition, the diffusion constant and activation energy of diffused gallium atoms were also obtained. Compared with titanium diffusion, gallium diffusion has a similar diffusion constant, but the activation energy is smaller, which means that the required diffusion temperature and time are relatively low and shorter. The designed 3-dB coupler proves the applicability of the waveguide, as shown in Figure 10. This work provides a feasible alternative for single-polarization applications and offers better compatibility with integrated applications on GaN-related LiNbO_3_ substrates. It provides a new and effective gallium diffusion method for the fabrication of LiNbO_3_ optical waveguides and offers potential applications in integrated optics and optoelectronic devices.

**Figure 11:**
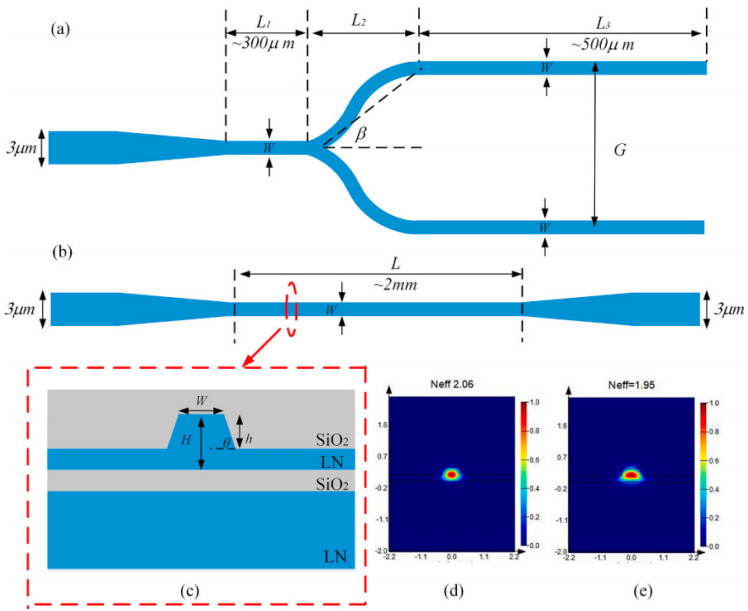
Configuration of (**a**) a cosine-shaped Y-branch optical waveguide and (**b**) a straight optical waveguide. (**c**) Z-cut TFLN ridge waveguide schematic. (**d**) Electromagnetic field distribution of the TE mode in a cross-section of the Z-cut TFLN ridge waveguide, where the effective refractive index is 2.06. (**e**) Electromagnetic field distribution of the TM mode in a cross-section of the Z-cut TFLN ridge waveguide, where the effective refractive index is 1.95. Reprinted/adapted with permission from Ref. [32]. 2021, Elsevier.

Ying Li et al. [32] prepared a low-loss ridge waveguide of thin-film LiNbO_3_, as shown in Figure 11. The waveguide was fabricated with a Z-cut LiNbO_3_ substrate using Smartcut technology. Proton exchange wet etching (PEWE) technology was used to achieve a better vertical sidewall angle and improve surface roughness, further reducing scattering loss. Through the improved proton exchange process, the average etching rate was significantly increased, and the final optimized etching rate reached 13.5 nm/min. With an 850 nm single-mode laser as the light source, the measured propagation loss of the linear ridge waveguide was 4.3 dB/cm. At the same time, a Y-shaped branch waveguide was also measured, and its optical splitting ratio was close to 1:1, which suggests high process compatibility. This study not only improves the etching rate but also does not damage the waveguide surface, achieving a quasi-vertical sidewall, which provides the possibility for the efficient manufacture of low-loss and ultra-compact ridge waveguide devices in the future. The above two optimized waveguide preparation methods reduce the transmission loss of LiNbO_3_ waveguides and are also applicable to heteroepitaxial thin films. The multilayer structure of the waveguide is very suitable for epitaxial preparation. Compared with the thin-film waveguide prepared by Smartcut technology, the other preparation methods in this section are simpler.

LiNbO_3_ optical waveguides encounter several critical challenges in the pursuit of high-performance photonic integrated devices. To mitigate loss within the optical waveguide, it is typically necessary to employ a larger waveguide bending radius and wider electrode spacing, which will increase the device size and increase the half-wave voltage of the modulator [135]. In addition, in the preparation process of a thin-film LiNbO_3_ optical waveguide, it is necessary to solve the preparation problem of a straight waveguide with a high aspect ratio and low optical loss and a curved waveguide with a large radius of curvature [136,137]. Therefore, enhancing the conversion efficiency, tunability and operating band expansion remain key challenges that need to be addressed in the study of LiNbO_3_ waveguides.

### 4.4. TFLN Electro-Optic Modulators

TFLN EOMs are usually composed of TFLN, an electrode and an optical waveguide. TFLN is prepared on the substrate material by epitaxial or wafer bonding processes, and an optical waveguide structure is constructed on it to guide the propagation of optical signals [138]. The metal electrodes are arranged on the surface of the LiNbO_3_ film to apply an external electric field. When the optical signal passes through the LiNbO_3_ waveguide, the applied electric field causes a change in the refractive index of the material, thereby modulating the phase or intensity of the optical signal [139].

EOMs can be divided into two types: phase modulators and intensity modulators. Phase modulators are usually used in coherent communication systems by changing the phase of the optical signal [140]. The intensity modulator transmits information by changing the intensity of the optical signal [141]. EOMs based on TFLN not only have the advantage of high modulation rates but also can work in a wide wavelength range, which allows them to occupy an important position in high-speed optical communication systems.

The EO coefficient of LiNbO_3_ material is exceptionally high. This remarkable property enables TFLN EOMs to achieve high-speed modulation with a low driving voltage, thereby offering significant advantages in the realm of high-speed and low-power optical communication [142]. In addition, the nonlinear optical properties of TFLN are also very suitable for integrated nonlinear optical devices, such as frequency converters and parametric amplifiers [143]. With the progress in manufacturing technology, the miniaturization and integration of TFLN devices is becoming possible. Through the new wafer bonding and ion-cutting process, high-quality TFLN can be prepared on common substrate materials such as silicon to integrate TFLN devices into the existing silicon photonic platform [142]. This heterogeneous integration technology helps to develop more complex and more efficient photonic integrated circuits [144].

**Figure 12:**
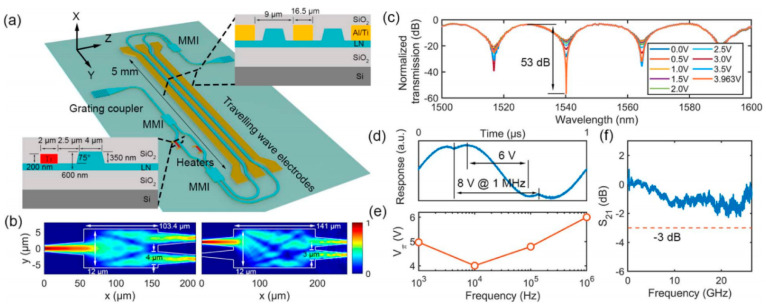
(**a**) Cascaded-MZI EOM schematic, including the cross-section of the thermo-optic structure in the first MZI and the EO structure in the second (modulated) MZI. (**b**) Electric field amplitude distributions of 1 × 2 MMI (left) and 2 × 2 MMI (right) were used in the modulator. (**c**) Measurement of the transmission spectrum of the heater under different DC voltages. (**d**) EO response driven by a 1 MHz frequency triangular wave. (**e**) The relationship between Vπ and driving frequency is measured. (**f**) The EO bandwidth (S_21_) of EOM is measured [145].

Yuan Shen et al. [145] designed an EOM based on TFLN to generate ultra-high-extinction-ratio optical pulses for distributed acoustic sensing, as shown in Figure 12. The electro-optic modulator is fabricated by electron beam lithography and inductively coupled plasma-reactive ion etching on a Z-cut LiNbO_3_ substrate fabricated using Smartcut technology. The researchers found an interface carrier effect on the TFLN EOM, resulting in a relaxation tail response during pulse modulation, and they solved this problem by adding a small compensation component after the main drive signal. Through the cascaded Mach–Zehnder interferometer (MZI) structure, an ultra-high extinction ratio of more than 50 dB was achieved. In addition, when the extinction ratio was increased from 20 dB to 50 dB, the spatial crosstalk suppression along the fiber reached 24.9 dB, which highlights the importance of the extinction for to the sensing performance. This work demonstrates the potential of TFLN EOMs in achieving ultra-high extinction ratio modulation and also provides a new direction for the development of optoelectronic modules for compact, low-power sensing systems.

**Figure 13:**
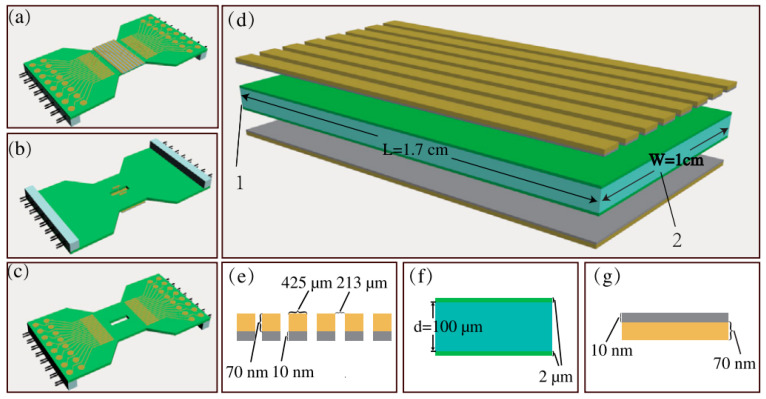
(**a**,**b**) Top and bottom of the LiNbO_3_-SLM structure, respectively. (**c**) Supporting device for clamping the electrode–LiNbO_3_ crystal. (**d**) LiNbO_3_ film with the electrode, which consists of three parts: the upper electrode layer, the intermediate crystal layer and the bottom electrode layer. The detailed structures of these three parts are shown in (**e**–**g**), respectively. Reprinted/adapted with permission from Ref. [36]. 2021, Optical Society of America.

As shown in Figure 13, Xuanchao Ye et al. [36] designed a high-speed programmable spatial light modulator based on a LiNbO_3_ film using Smartcut, which realized low driving voltage (10 V) and high-speed modulation up to 5 MHz by using the EO effect. Compared with traditional liquid crystals, digital micromirror devices and microelectromechanical system spatial light modulators, the modulator exhibits a significant improvement in response speed and can meet the needs of various high-speed applications such as optical communication, imaging and video projection. An image transmission experiment using this modulator proved its potential in parallel data transmission. In addition, the modulator has the advantages of simple design; flexible switching between phase, polarization and amplitude modulation modes; a high optical damage threshold; an ultra-wide transparent window; and ultra-low optical loss, which opens up new possibilities for high-speed real-time applications such as LiDAR, pulse shaping and beam steering. This new EOM preparation method is very suitable for PLD and magnetron sputtering technology. The film and electrode prepared by sputtering are more closely bonded, which reduces the transmission loss, and the compensation of the substrate can effectively improve the response speed of the device.

TFLN EOMs still have shortcomings in some aspects. The preparation and processing technology of LiNbO_3_ is relatively complex and costly. In particular, the preparation of high-quality thin films still depends on high-precision epitaxial growth or wafer bonding technology, which makes LiNbO_3_-based devices costly and limits their popularity in low-cost application scenarios [146]. In addition, although LiNbO_3_ films can be integrated onto silicon substrates by wafer bonding, the thermal stability and matching problems of this heterogeneous integration still need to be further solved to ensure the reliability of these devices in the high-temperature working environment.

### 4.5. TFLN Microphotonic Devices

TFLN is not only effective for EOMs but also holds significant potential in the realm of microphotonic devices. Microphotonic devices refer to optical devices with a size of microns or even nanometers, which are usually used in photonic integrated circuits to realize the manipulation and processing of light. LiNbO_3_ has become an ideal material for microphotonic devices due to its excellent EO effect and nonlinear optical properties [111].

In microphotonic devices, TFLN is usually used in combination with photonic crystals [147], microring resonators [148] and other structures. The microring resonator is a typical microphotonic device that realizes the storage and regulation of light in the microring structure through the resonance effect of light. When a LiNbO_3_ film is combined with a microring resonator, the applied electric field can change the refractive index of the LiNbO_3_ film, thereby regulating the resonant frequency of the microring resonator and achieving accurate modulation of the optical signal. This combination provides TFLN-based microphotonic devices with higher modulation depth and lower power consumption, which is especially suitable for high-speed photonic integrated circuits [47]. In addition, TFLN can also be combined with other nonlinear optical structures and applied to optical frequency conversion, optical parametric amplification and other fields. By integrating multiple functional devices, highly integrated photonic chips can be realized to meet the needs of modern optical communication systems for high speed, large capacity and low delay.

**Figure 14:**
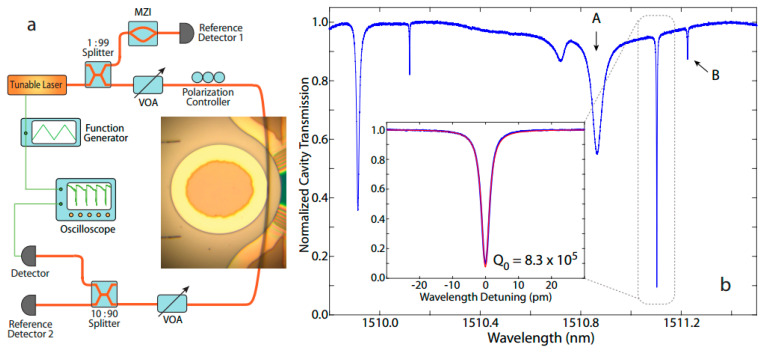
(**a**) Schematic diagram of the experimental device, where the insert shows the optical microscopic image of the device coupled to the tapered fiber for light entry and exit equipment. (**b**) Laser scanning quasi-TE polarized cavity transmission spectrum [149].

Xuan Sun et al. [149] studied the nonlinear optical oscillation dynamics in high-Q LiNbO_3_ microresonators. It was found that the unique competition between thermo-optical nonlinearity and the photorefractive effect led to complex nonlinear optical behavior, which had not been reported in previous studies or mechanisms. As shown in Figure 14, an X-cut LiNbO_3_ device with a thickness of 300 nm on a 2 μm thick silica substrate was fabricated using Smartcut technology, and its nonlinear optical behavior was explored by applying continuous wave laser input and monitoring the time-dependent waveform of light waves. With an increase in the input optical power, the resonator exhibits a typical bistable behavior and the resonance position of the resonant cavity moves to a short wavelength, which is different from the conventional thermo-optical bistable behavior. In addition, when the laser wavelength is shifted from red to blue, the resonant cavity transmission spectrum begins to broaden and finally shows significant oscillation. This study successfully describes the experimental phenomena through theoretical models and provides important insights for understanding the nonlinear optical behavior of high-Q LiNbO_3_ microphotonic devices, which is of great significance for the future application of nonlinear LiNbO_3_ photonics in integrated photonics.

**Figure 15:**
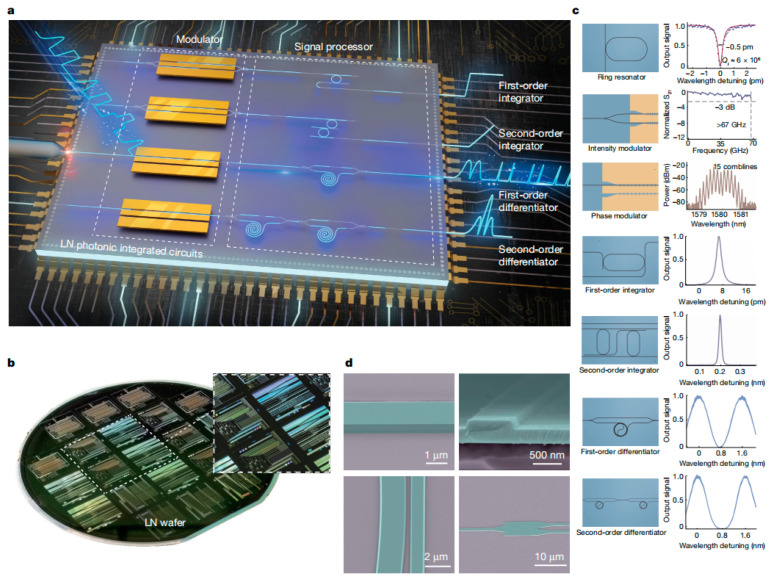
Wafer-level LiNbO_3_-based MWP signal-processing engine and its components. (**a**) Schematic diagram of the LiNbO_3_-based MWP processing engine consisting of a high-speed EO modulation module and a low-loss, multi-purpose photon processing part. (**b**) The 4-inch wafer-level LiNbO_3_ photonic integrated circuit was patterned using an ultraviolet step lithography system. (**c**) Microscopic image and key performance indicators of the basic components of the high-speed MWP system. (**d**) False-color scanning electron micrographs of the device, showing the side wall of the waveguide, the coupling region of the microresonator, the cross-section view of the waveguide and the multimode interference coupler. Reprinted/adapted with permission from Ref. [150]. 2024, Springer Nature.

Hanke Feng et al. [150] developed an integrated microwave photonic (MWP) processing engine based on a 4-inch wafer-scale TFLN platform, as illustrated in Figure 15. This engine achieves a processing bandwidth of up to 67 GHz under complementary metal-oxide semiconductor (CMOS)-compatible voltages, offering a chip-level optical system for the generation, transmission and manipulation of microwave signals. The platform not only features an efficient high-speed electro-optic modulator (EOM) but also integrates a low-loss functional photonic network capable of performing various signal-processing tasks. The engine excels in ultra-fast simulation calculations such as time-domain integration and differentiation, with a sampling rate of up to 256 giga-samples per second. Additionally, the platform is employed for solving ordinary differential equations, generating ultra-wideband signals and detecting edges in images for conceptual verification applications. Notably, the researchers utilized the platform’s image edge detection capabilities to effectively segment melanoma lesion boundaries in medical diagnostic images, highlighting its potential in photon-assisted image segmentation models. The advent of this integrated MWP engine provides a compact, low-latency and cost-effective solution for future wireless communication, high-resolution radar and photonic artificial intelligence. The microphotonic integration platform based on large-size TFLN has shown great potential in the fields of high-speed optical communication, quantum information processing, microwave photonics, nonlinear optics and sensing due to the advantages of a high electro-optic coefficient, wide transparent window and low loss characteristics. Although most large-size LiNbO_3_ films are obtained using Smartcut technology in terms of material growth, with the progress in film preparation technology, advances are expected to enable epitaxial LiNbO_3_ integrated chips and promote the development of optoelectronic integrated chips.

Future research endeavors should concentrate on enhancing the integration and compatibility of TFLN devices so that they can be better integrated into the existing silicon photonic technology platform [151]. Through heterogeneous integration with silicon photonic devices, more competitive photonic chips can be developed to achieve high-speed, low-power optical communication and information processing [152]. With the ongoing advancements in manufacturing and integration technologies, TFLN devices are poised to play an increasingly critical role in the future of photonic integrated circuits [153].

## 5. Conclusions and Prospects

TFLN shows great application potential in the field of optical communication and photonic integrated circuits due to its excellent ferroelectric, piezoelectric and photoelectric properties. In this review, various preparation methods of TFLN are discussed, including CVD, MBE, PLD, magnetron sputtering and Smartcut technology. The unique advantages and technical optimization measures of TFLN prepared using each method are introduced in detail. Epitaxial thin films can be compatible with semiconductor processes, facilitating integration into existing electronic and optoelectronic devices and promoting the development of a new generation of high-performance and miniaturized devices. Therefore, the development of LiNbO_3_ epitaxial films is of great significance for promoting the progress of optoelectronic technology and integrated optical systems. In the field of device applications, TFLN, with its excellent electro-optical, acousto-optical and nonlinear optical properties, will shine in the fields of optical communication, optical computing, quantum information and so on. Its nonlinear optical effect can enable the fast processing and operation of optical signals. With the maturity of thin-film preparation technology and the reduction in cost, epitaxial TFLN is expected to achieve breakthroughs in emerging fields and become an important supporting material for future optoelectronic technology. TFLN devices have the advantages of miniaturization, high performance and integration and have become an indispensable part of photonic integrated circuits.

With the continuous advancement of manufacturing technology and integration technology, TFLN will show broad application prospects and huge market potential in the fields of integrated optics, quantum communication and high-frequency electronic devices. The focus of research will be on improving the integration and compatibility of devices and developing heterogeneous integration with silicon-based technology to achieve higher speed and lower power consumption in optical communication and information processing. Epitaxial TFLN has a low production cost, and optimizing its processing technology will be an important direction for future research. First of all, by using cheap and stable precursors (metal–organic compounds or inorganic salts), the cost of materials can be reduced, and the utilization rate of materials can be improved. In terms of equipment, the development of multi-chamber reactors or batch deposition processes can improve production efficiency and reduce the production cost of single films. In addition, optimizing the process parameters (airflow, temperature and pressure) to ensure film thickness uniformity and a low defect rate will help reduce the complexity and cost of subsequent processing and pave the way for large-scale industrial applications.

## Figures and Tables

**Table 1:** Optimization of LiNbO_3_ films prepared by CVD.

Publishing Year	Precursor Material	Precursor Optimization Measures	Optimization of Instrument Preparation Method
1993 [65]	Niobium(V) ethoxide, lithium dipivaloylmethanate (LiDPM)	The formation of bimetallic complexes by the reaction of niobium(V) ethoxide and LiDPM optimized the volatility and thermal stability of the precursor.	Spray MOCVD technology was used to optimize the volatilization and deposition process of the precursor through two temperature regions (evaporation region and deposition region) in the hot-wall reactor.
1995 [66]	Lithium tert-butoxide [Li(O^t^Bu)], niobium ethoxide [Nb(OEt)_5_]	Using Li(O^t^Bu) as the lithium source, the deposition temperature was reduced to 450 °C, and the volatility and reaction conditions of the precursor were optimized.	Using a low-pressure MOCVD system, the transport and deposition process of the precursor was optimized through a quartz reaction tube and a stainless steel vessel.
2009 [61]	Li(O^t^Bu), niobium tetra-ethoxy di-methyl-amino-ethoxide (Nb(OEt)_4_(dmae))	The effects of different precursor ratios and total flow rates on the properties of the films were studied by optimizing the precursor ratio using a combination of chemical methods.	The linear gradient transport and deposition process of the precursor was optimized via pre-chamber design using a Sibylla-150 high-vacuum CVD reactor (ABCD Technology Sarl, Geneva, Switzerland).
2013 [67]	Li(thd), Nb(thd)_4_ (thd = 2,2,6,6-tetramethyl-3,5-heptanedionate)	The precursor ratio and deposition pressure were optimized, and their effects on the phase composition, Li nonstoichiometric ratio, texture, epitaxial quality and twin formation of the films were studied.	Using pulsed-injection MOCVD and atmospheric-pressure MOCVD systems, the precise control and uniform deposition of precursors were optimized through electromagnetic injectors and ultrasonic atomizers.

**Table 2:** Optimization of LiNbO_3_ films prepared by MBE.

Publishing Year	Precursor Material	Precursor Optimization Measures	Optimization of Instrument Preparation Method
1985 [24]	Niobium (Nb), lithium (Li), oxygen (O_2_)	An electron beam evaporator was used to heat niobium and lithium to ensure a high evaporation temperature (about 2400 °C for Nb).	The vacuum environment was optimized using a dual-cavity system, and in situ monitoring was performed using reflective high-energy electron diffraction (RHEED).
1999 [74]	Lithium niobate (LiNbO_3_) target, O_2_	Laser molecular beam epitaxy (L-MBE) was performed using a KrF excimer laser (248 nm) to introduce an oxygen radical source to improve oxygen reactivity.	Using low background pressure (1.3 × 10^−5^ Torr), RHEED was used for in situ monitoring, and laser pulse energy and frequency were optimized.
2008 [79]	Cobalt-doped lithium niobate target, O_2_	The target was doped with Co to increase the atmospheric pressure of oxygen (10 Pa).	The distance between the target and the substrate (5 cm) was optimized.
2017 [80]	Niobium chloride (NbCl_5_), Li, O_2_	NbCl_5_ was used as a high-vapor-pressure Nb precursor, and Li was used as a chlorine capture agent to optimize the flow ratio from Li to NbCl_5_.	High-temperature (800–1050 °C) growth was used to optimize the oxygen flow rate (4 sccm). The structure was characterized by RHEED and XRD.

**Table 3:** Optimization of LiNbO_3_ films prepared by PLD.

Publishing Year	Optimization Measures	Optimization of Instrument Preparation Method
1995 [94]	A Li enrichment target was used to ensure the stoichiometric ratio of the lithium niobate film. The laser energy density and gas pressure were optimized to reduce Li loss.	An ArF excimer laser (193 nm) was used, and the substrate temperature was controlled at 500–800 °C. The samples were deposited in an O_2_ or O_2_/O_3_ mixed-gas environment.
1995 [95]	The effects of oxygen pressure and laser energy density on the optical properties and composition of the films were studied. The stoichiometric ratio of the film was optimized by using an Li-enriched target, and the film growth process was monitored by real-time reflectivity.	An ArF excimer laser (193 nm) was used for deposition in the oxygen pressure range of 10^−5^–10^−1^ mbar. A He-Ne laser was used to monitor the reflectivity of the film in real time.
2000 [96]	Low-electric-field-assisted deposition was used to optimize the crystal orientation of the film. The substrate temperature and oxygen pressure were optimized to reduce Li loss. Oxygen vacancies in the film were reduced by annealing.	A KrF excimer laser (248 nm) was used. The substrate temperature was controlled at 550–800 °C. Deposition was performed in an oxygen atmosphere with annealing for 30 min after deposition.
2017 [97]	The thickness and surface roughness of the film were optimized by dynamic theory simulation. The effect of the plasma expansion process on the film growth mode was studied.	Numerical simulation was carried out by using Matlab software (https://ww2.mathworks.cn/en/products.html, accessed on 10 February 2025). The laser energy density and the distance between the target and the substrate were further optimized to improve the film quality.
2018 [98]	The effects of laser pulse number on the thickness and electrical properties of the films were studied. The grain size and carrier mobility of the films were optimized.	A KrF excimer laser (248 nm) was used. The substrate temperature was controlled at 300–600 °C and deposited in an Ar atmosphere, which further reduced the roughness of the film.

**Table 4:** Optimization of LiNbO_3_ films prepared by magnetron sputtering.

Publishing Year	Optimization Measures	Optimization of Instrument Preparation Method
1984 [104]	The substrate cleaning method was optimized by using discharge cleaning and solvent degreasing to enhance the adhesion between the film and the substrate. A LiNbO_3_ powder target was used to avoid the fracturing of the single-crystal target due to local heating.	The substrate temperature was controlled at 380–470 °C. Liquid nitrogen was used to remove water vapor to prevent film absorption.
2001 [105]	The sputtering parameters were optimized, including RF power, gas pressure, substrate temperature, etc., to ensure the stoichiometric ratio of the film. The structure and interface quality of the films were analyzed by XRD and TEM.	An RF magnetron sputtering system was used with a frequency of 13.56 MHz. The substrate temperature was controlled at 490 °C. An Ar/O_2_ gas mixture was used at a pressure of 30 mTorr. A multi-step sputtering process was developed to gradually increase the thickness of the film to ensure the structural and optical properties of the film.
2017 [106]	The initial growth stages of TFLN on different substrates were studied, and the sputtering conditions were optimized. Through the plasma effect, the growth quality of the film was improved.	The substrate temperature was controlled at 550 °C. Ar gas was used at a pressure of 0.8 Pa. The position of the substrate and the target was optimized to ensure a uniform plasma effect.

**Table 5:** Advantages, disadvantages and future research directions of TFLN preparation methods.

	Advantages	Disadvantages	Optimization Directions
CVD	High deposition rate, large-area film preparation, precise control of film thickness and composition.	Most of the precursors are toxic, easy to introduce impurities.	Develop new precursors with low toxicity and high stability. Optimize the deposition conditions to improve the quality and uniformity of the film.
MBE	Precise control of growth, growth in an ultra-high-vacuum environment, high film quality, growth process can be monitored in real time.	Lithium is easy to volatilize, the composition of the film deviates from the stoichiometric ratio, a multiphase structure is formed.	Optimize the growth parameters (substrate temperature, lithium/niobium flow ratio), develop new precursor materials to increase the growth rate.
PLD	Thin-film epitaxial growth, high crystal quality, good uniformity, impurity-free atom introduction.	Slow film growth rate, small film area, difficult for large-scale industrial production.	Optimize the deposition parameters (substrate temperature, laser pulse frequency), explore new target and substrate materials.
Magnetron sputtering	Fast deposition rate, large film area, suitable for industrial production.	The uniformity of the large-size film thickness is poor.	Optimize sputtering parameters and substrate temperature control, develop new sputtering process to improve the quality and uniformity of the film.
Smartcut	High crystal quality, the film is easy to transfer, suitable for heterogeneous integration.	Ion implantation can easily introduce defects. Processing requires high-precision equipment and process control, resulting in high production costs.	Accurately control the depth of ion implantation, reduce lattice damage, optimize manufacturing techniques.
